# Research advances in chikungunya virus: Epidemiology, pathogenesis, and control

**DOI:** 10.1016/j.onehlt.2026.101413

**Published:** 2026-04-15

**Authors:** Shiqin Dai, Hao Li, Shu Li, Xinhua Shao, Xianliang Cheng

**Affiliations:** a The People's Hospital of Longhua, Shenzhen, Guangdong, China; b German Cancer Research Center (DKFZ) Unit D400, Heidelberg, Germany; c Department of Vascular Biology, European Center for Angioscience (ECAS), Medical Faculty Mannheim, Heidelberg University, Mannheim 68167, Germany; d Center for Endemic Disease Control, Chinese Center for Disease Control and Prevention, Harbin Medical University, Harbin, Heilongjiang 150081, China; e Zigong Academy of Medical Sciences, Zigong First People's Hospital, Zigong, Sichuan 643000, China; f Medical Faculty, Heidelberg University, Heidelberg, Germany; g Institut National de la Santé et de la Recherche Médicale (INSERM) mixed Unit, Heidelberg, Germany

**Keywords:** Chikungunya virus, CHIKV, *Aedes aegypti*, *Aedes albopictus*, Vaccine development

## Abstract

Chikungunya virus (CHIKV) is a mosquito-borne RNA virus endemic to tropical regions, where it causes recurrent outbreaks. Transmitted primarily by *Aedes aegypti* and *Aedes albopictus* mosquitoes, CHIKV has expanded from its origins in Africa to affect millions across Asia, the Americas, and East and Central Africa in recent decades. Infection results in chikungunya fever, a debilitating illness marked by acute fever and severe musculoskeletal symptoms—including persistent joint and muscle pain. These symptoms often continue for years, impairing health, quality of life, and economic productivity. Although CHIKV pathogenesis has been studied for more than half a century, no specific antiviral treatment exists to date. Clinical management remains supportive, focused on relieving symptoms and improving patient comfort. In light of its continued global spread, advancing the understanding of CHIKV pathogenesis is urgently needed. Such insights are essential for elucidating disease mechanisms and facilitating the development of effective preventive and therapeutic interventions. This review systematically summarizes the latest advances in CHIKV research, covering its epidemiology, transmission, viral replication, clinical manifestations, immunopathology, diagnosis, vaccine and antiviral development, and integrated control measures, while identifying key knowledge gaps and unmet public health needs. From a One Health perspective, it clarifies human-vector-environment links in CHIKV transmission, informs cross-disciplinary collaboration and coordinated control, advancing One Health practice against mosquito-borne diseases.

## Highlights

• 2024–2025 CHIKV epidemiology, outbreaks and genotype evolution in China Asia.
• Molecular mechanisms and inflammatory pathways of CHIKV chronic arthralgia.
• Ixchiq clinical application and latest CHIKV vaccine/antiviral advances.
• One Health CHIKV surveillance system with human-veterinary-environmental data.

## 1 Introduction

Arboviruses, including yellow fever, dengue, Zika, and chikungunya, pose a significant public health threat in tropical and subtropical regions, transmitted to humans through mosquito vectors. Among these, the arthritogenic alphaviruses represent a major cause of debilitating polyarthralgia worldwide. The most prominent of these, the chikungunya virus (CHIKV), constitutes a substantial and growing global health threat. The CHIKV first identified in 1952 on the Makonde Plateau in Tanzania, is a mosquito-borne pathogen that has evolved into a significant global health concern. Following its initial isolation, CHIKV was soon identified in Asia. The first clinical case was confirmed in India in 1954, followed by the first laboratory-confirmed outbreak in Thailand in 1958. For the next three decades, from the 1960s to the 1980s, sporadic outbreaks were reported across Africa and Asia. A major pandemic between 2004 and 2007 marked a turning point, with over 1.5 million cases reported across 60 countries. The virus has since expanded into Europe and the United States, demonstrating its capacity for widespread transmission. As of December 2024, local transmission of CHIKV has been documented in 119 countries and territories, with the highest burden concentrated in the Americas, Asia, and Africa. In the first half of 2025 alone, approximately 220,000 cases and 80 deaths were reported across 14 affected countries and regions [1].

CHIKV has established a stable human-amplified transmission cycle, primarily driven by the invasive *Aedes aegypti* and *Aedes albopictus* mosquitoes. This threat is amplified by the virus's continuous evolution. Among the three major genotypes—West African, East/Central/South African (ECSA), and Asian—the ECSA lineage, in particular, has accumulated specific mutations that enhance its fitness and transmissibility [2]. This persistent and expanding geographic footprint is evidenced by its staggering annual infection rate of an estimated 16.9 million people, placing nearly 2.8 billion individuals in at-risk regions in danger [3].

After an infected person is bitten by a mosquito, the virus requires 2–10 days to incubate within the mosquito before it becomes capable of transmitting the infection to other humans through subsequent bites [4], [5], [6]. Clinically, chikungunya infection is characterized by acute fever and severe joint pain. While the case fatality rate is relatively low (approximately 0.3%), a significant proportion of symptomatic patients (44%) progress to a chronic phase of the disease [7]. The clinical manifestations primarily include fever, rash, and joint muscle pain. While most patients improve within one week, approximately 30%–40% may experience persistent joint pain lasting months to years, significantly impacting long-term quality of life [8]. The long-term burden of the disease falls hardest on vulnerable groups, with infants and the elderly being most at risk for serious neurological problems [2], [8].

The pursuit of vaccines against CHIKV has leveraged diverse technological platforms, including formalin-inactivated whole virus, live-attenuated strains, virus-like particles (VLPs), viral vector constructs, and mRNA-based candidates. Despite these efforts, the absence of licensed vaccines or specific antivirals against CHIKV continues to leave populations susceptible to recurrent outbreaks and long-term sequelae, highlighting a persistent and urgent public health gap. Notably, existing reviews have predominantly focused on isolated aspects of CHIKV (e.g., single vaccine platforms or acute-phase pathogenesis) and lack a comprehensive integration of One Health principles—particularly the interconnectedness of sylvatic-urban transmission cycles, vector adaptation, and cross-species spillover risks. Furthermore, critical knowledge gaps remain unaddressed, such as the genotype-specific clinical outcomes, mechanisms of chronic arthralgia, and translational barriers for antiviral development. To fill these gaps, this review systematically synthesizes the latest advances in CHIKV research (2020–2025) across epidemiology, transmission, pathogenesis, diagnosis, and control, with a core focus on integrating sylvatic cycle surveillance data (e.g., non-human primate seroprevalence) with urban transmission dynamics, critically comparing genotype-specific adaptations (e.g., E1-A226V in ECSA lineage) and their impacts on vector competence and clinical severity, and identifying translational bottlenecks in vaccine/antiviral development to propose actionable public health strategies for endemic and non-endemic regions. By addressing these aspects, this review provides a holistic and critical perspective that advances beyond descriptive summaries to guide future research and policy.

## 2 Epidemiology and transmission dynamics of chikungunya virus

**Fig. 1:**
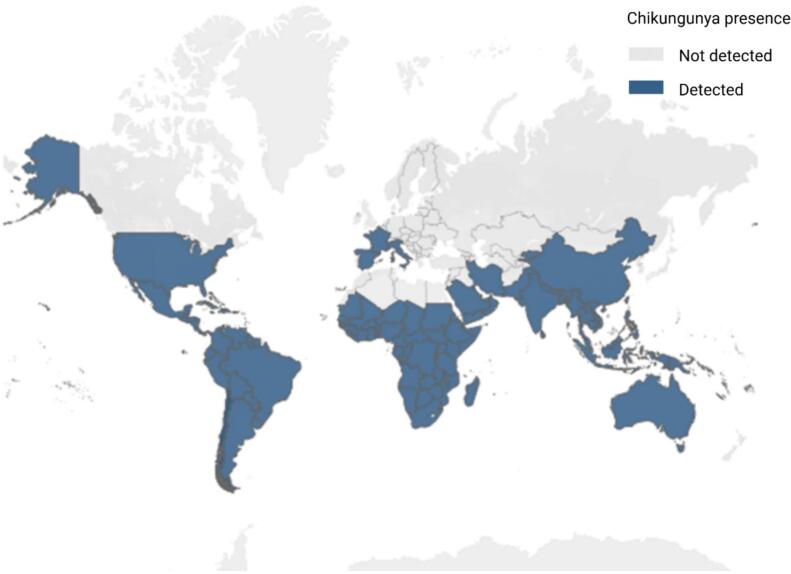
Global distribution and transmission of CHIKV.

From its origins in East Africa in 1952, CHIKV has emerged as a pathogen of growing international concern, with its most dramatic global expansion occurring over the last two decades. A large epidemic in Kenya spread to La Réunion Island in 2005, and because of a mutation that allowed more-efficient transmission by *Aedes albopictus*, large epidemics occurred among islands in the Indian Ocean, India, Southeast Asia, and countries in Africa, with some spread in Europe. Near the end of 2013, CHIKV was introduced to the Caribbean and resulted in a large outbreak that has now spread to Mexico and many countries in Central and South America. Brazil has experienced multiple outbreaks of CHIKV and currently has the highest cumulative number of confirmed cases globally. It is followed by China, Bolivia, Argentina, and India. Other countries and regions reporting cases are primarily characterized by sporadic infections, with overall relatively low prevalence levels. These countries and regions have all reported locally transmitted cases, with some areas experiencing a rapid increase in case numbers, even accompanied by reported deaths. Globally, the number of chikungunya fever cases has shown a gradual increasing trend since 2017. Among these, CHIKV situation in the Americas has risen rapidly since 2018, with case numbers increasing year by year, reaching a peak during 2023–2024 (with over 400,000 infected cases). The region has now become the most severely affected area, exhibiting typical endemic characteristics, particularly in countries such as Brazil, Paraguay, and Argentina [9]. Local transmission of chikungunya has been documented in 119 countries and territories. However, global epidemiological data remain incomplete and often untimely, as many affected nations face challenges in surveillance, case detection, diagnostic capacity, and reporting. Consequently, the absence of reported cases should not be misinterpreted as evidence that transmission is absent (Fig. 1).

Currently, the geographical distribution of CHIKV is characterized by its endemic presence in tropical or subtropical regions, including sub-Saharan Africa, Southeast Asia, the Indian Ocean coast and islands, and the Western Pacific. This spread is largely attributed to globalization-driven travel and trade, as well as the northward expansion of *Aedes* mosquitoes into more temperate zones due to climate change. Notably, the year 2024 to the first half of 2025 has witnessed a growing public health challenge posed by imported CHIKV cases in non-endemic regions of Asia, especially in southern China. Cities including Foshan and Guangzhou have reported sporadic local outbreaks linked to imported infections, which have raised substantial prevention and control pressure. Such local transmission events are predominantly driven by the high adaptability of *Aedes albopictus* to urban microclimates in these regions: the mosquito species has evolved to thrive in artificial urban habitats such as water-accumulating containers in residential areas, construction sites and public green spaces, and its tolerance to mild temperature fluctuations and low humidity in urban environments has further enhanced its vector competence and transmission efficiency of CHIKV in densely populated urban settings [10], [11], [12]. Phylogenetic analysis confirmed the 2025 Foshan outbreak strain belongs to the ECSA lineage (MAL), and all circulating strains harbored the E1-A226V adaptive mutation (along with E2-I211T) [13]. This single amino acid substitution in the E1 envelope protein alters the conformation of the viral fusion loop, significantly enhancing viral attachment, midgut epithelial cell infection, and dissemination efficiency in *Aedes albopictus*—the dominant regional vector in South China (Foshan/Guangzhou) and temperate/subtropical Asia [14]. The E2-I211T mutation further potentiates the transmission advantage of E1-A226V by providing a favorable genetic background, which together drives the high transmissibility of the ECSA lineage in *Aedes albopictus*-dominant regions and directly facilitated the 2025 local outbreak in Foshan linked to imported cases [13]. In particular, *Aedes albopictus* has adapted to colder environments, further facilitating the global expansion of CHIKV.

Sylvatic transmission cycles remain pivotal reservoirs for CHIKV spillover, with non-human primates (NHPs) acting as primary enzootic hosts that sustain viral circulation in natural ecosystems. In Africa, CHIKV is maintained through a well-established sylvatic cycle involving diverse NHP species and forest-dwelling mosquito vectors (e.g., *Aedes africanus*, *Aedes furcifer*). Serological surveillance across sub-Saharan regions confirms a pooled CHIKV seroprevalence of 35% (95% CI: 9–66%) in African NHPs, a finding that reflects persistent enzootic activity across forested landscapes. Periodic spillover to humans in rural and forest-urban interface areas is driven by cycling herd immunity among NHPs, as empirically documented in Senegal and other endemic regions. In Southeast Asia, recent surveillance efforts have increasingly validated active sylvatic circulation: serological investigations in Thailand detected CHIKV-neutralizing antibodies in wild macaques across multiple national parks, while a 2022 cross-sectional study in peri-urban Yangon (Myanmar) identified 33% CHIKV seroprevalence in NHPs without concurrent human cases—providing direct evidence of enzootic transmission independent of urban spillback. Collectively, these data underscore NHPs as key sentinels for hidden enzootic transmission, with high spillover risks concentrated in peri-urban forest edges, tourist sites, and deforested areas where human-NHP contact is frequent. To address this risk, NHP serosurveillance has been integrated into the revised “One Health” surveillance framework as a core module, with a predefined threshold trigger (>10% seroprevalence in peri-urban NHPs) to initiate targeted vector control and public health alerts—directly enhancing spillover risk assessment at the human-wildlife interface [15]. While a sylvatic (forest) transmission cycle persists in Africa and parts of Asia, the urban human-mosquito cycle has become epidemiologically dominant.

**Fig. 2:**
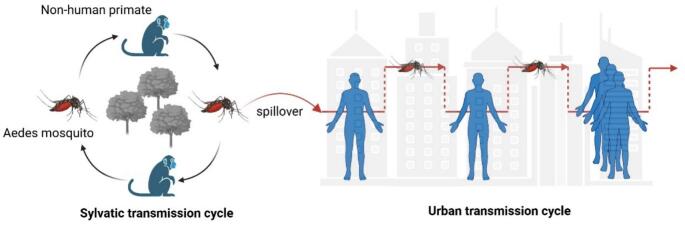
The sylvatic (forest) and urban transmission cycles of CHIKV.

In large populations, transmission can be sustained over time as a sufficient number of immunologically naive individuals become infected, thereby propagating further spread (Fig. 2).

## 3 Entry and replication of chikungunya virus

CHIKV belongs to the genus *Alphavirus* within the family *Togaviridae*. The viral particles are spherical, enveloped, and approximately 65 nm in diameter [16]. The genome consists of a positive-sense single-stranded RNA molecule, which is encapsidated by viral capsid proteins and further enclosed by a host cell-derived lipid envelope. This envelope is studded with viral envelope proteins that form an icosahedrally symmetric (*T* = 4) glycoprotein shell. The genomic RNA (gRNA) of CHIKV is approximately 12 kb in length and contains two open reading frames (ORFs) that encode the nonstructural polyprotein and the structural polyprotein, respectively. The gRNA within the viral particle structurally resembles cellular mRNA, featuring a 5′ cap and a 3′ polyadenylated tail [17]. This configuration allows immediate initiation of translation upon entry into the host cytoplasm [18].

The entry of CHIKV into host cells is primarily believed to occur through clathrin-mediated endocytosis following binding to cell membrane receptors. This mechanism is supported by the identification and in vivo validation of MXRA8 as a functional receptor for CHIKV and related arthritogenic alphaviruses in human cells [19]. MXRA8 is widely expressed in various tissues and can mediate infection in synovial fibroblasts, osteoblasts, chondrocytes, and skeletal muscle cells in vitro, indicating its important role in viral pathogenesis. Several potential attachment or entry co-factors have been proposed, including the widely expressed prohibitin, T-cell immunoglobulin and mucin domain protein 1 (TIM1), dendritic cell-specific intercellular adhesion molecule-3-grabbing non-integrin (DC-SIGN) [20], basigin (CD147), and glycosaminoglycans such as heparin and heparan sulfate. However, blocking MXRA8 in mice only partially reduced CHIKV, suggesting other attachment factors aid infection. No functional MXRA8 homolog exists in mosquitoes, and no definitive CHIKV receptor has been identified there, though proteins like ATP synthase β subunit and HSC70 may assist viral attachment.

Upon entry into the host cell, the 5′ open reading frame (ORF) of the viral genomic RNA (gRNA) is translated to produce four nonstructural proteins (nsP1, nsP2, nsP3, and nsP4), which are essential for viral genome synthesis and other replication-related functions [21]. These four proteins (nsP1 to nsP4) together form the replicase complex, which catalyzes the synthesis of new viral RNA. Within this complex, nsP4 functions as the RNA-dependent RNA polymerase and is responsible for polyadenylation, while nsP1, in coordination with nsP2, adds a cap structure (Cap-0) to the 5′ end of newly synthesized positive-sense viral RNAs [22].

The 3′ ORF, translated from a subgenomic RNA (sgRNA) transcribed from the negative-sense antigenome, encodes a polyprotein precursor (C–E3–E2–6 K/TF–E1). This precursor is cleaved into the structural proteins (Capsid, E1, E2, E3, 6 K, and TF), which form the viral particle and mediate virion assembly, budding, and host cell entry [16]. The capsid protein (C) is cleaved and released via its own autoprotease activity and remains in the cytoplasm. The envelope proteins, meanwhile, are processed and modified during their transit through the endoplasmic reticulum and the *trans*-Golgi network, ultimately being presented on the surface of the virion as trimers of E2–E1 heterodimers [15]. The E2 envelope subunit is the primary determinant of receptor binding, whereas the contains the fusion loops that mediate membrane fusion. During the infection of new cells, these fusion loops facilitate the merging of the viral envelope with the endosomal membrane, thereby releasing the viral RNA into the cytoplasm. The E3 envelope subunit serves a chaperone function by preventing the premature exposure of the E1 fusion loops prior to viral maturation. The 6 K/TF protein acts as an ion channel in the endoplasmic reticulum and contributes to the efficient assembly and budding of viral particles [23]. The functional roles and pathogenic mechanisms of CHIKV proteins are summarized in Table 1.

**Table 1:** Functional roles and pathogenic mechanisms of CHIKV proteins.

Protein type	Name	Function	Pathogenic association
Non-structural Proteins	nsP1	Viral RNA capping enzyme, involved in membrane anchoring	Replication complex assembly [25]
	nsP2	Helicase/protease activity, inhibits host interferon response (key immune evasion mechanism)	Triggers inflammatory storm [26]
	nsP3	Replication complex assembly scaffold	Viral replication efficiency [27]
	nsP4	RNA-dependent RNA polymerase (RdRp)	Genome replication
Structural Proteins	C	Capsid assembly, packages RNA genome	Nucleocapsid formation [28]
	E1	Membrane fusion protein, binds to host cell receptors	Critical for cell entry [29]
	E2	Receptor-binding domain, forms heterodimeric spikes with E1	Targets MXRAS receptor [18], [30]
	6 K	Ion channel, facilitates viral budding	Virus release

**Fig. 3:**
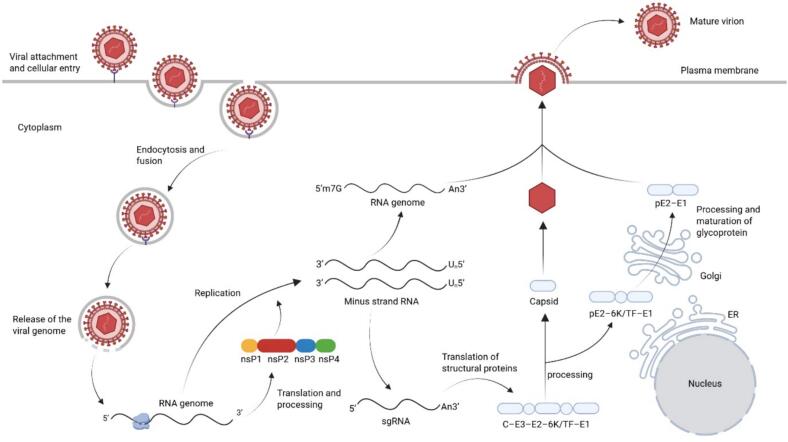
Viral infection is initiated through attachment to cellular receptors—including GAGs, MXRA8, DC-SIGN, and TIM—followed by internalization via clathrin-mediated endocytosis. Within the acidic environment of the endosome, conformational changes in the viral envelope—mediated by the E1 protein—trigger fusion between the viral and host membranes. This fusion results in the release of the nucleocapsid into the cytoplasm, where the viral genomic RNA (gRNA) is exposed and becomes accessible to the host translation machinery. The viral genome is translated into the non-structural polyprotein P1234, which is subsequently cleaved—primarily by the protease activity of nsP2—to produce the mature non-structural proteins nsP1–nsP4. The replicase directs the synthesis of a full-length negative-sense antigenome (-ssRNA), which in turn serves as the template for producing both new genomic ssRNA and subgenomic RNA (sgRNA). The sg RNA is translated at the rough endoplasmic reticulum into a structural polyprotein (C–pE2–6 K–E1). The capsid protein (C) rapidly autoproteolytically cleaves itself and is released into the cytoplasm, where it packages genomic RNA to form nucleocapsids. The remaining envelope polyprotein is inserted into the ER membrane and cleaved into pE2, 6 K, and E1. These proteins traffic to the Golgi apparatus and undergoes glycosylation. Mature E1 and E2 form heterodimers that organize into plasma membrane microdomains. Cytoplasmic nucleocapsids are recruited to these sites, leading to budding and release of mature virions.

Packaging of the viral genomic RNA (gRNA) depends on the specific binding of the capsid protein to a packaging signal sequence on the gRNA. Concurrently, the capsid protein interacts with the cytoplasmic tail of the E2 envelope protein, coordinating the envelopment of the nucleocapsid during the budding of nascent viral particles [24] (Fig. 3).

## 4 Clinical manifestations of chikungunya virus infection

CHIKV enters the host through the skin during blood feeding by an infected female mosquito. The virus is injected into the dermis along with mosquito saliva and may directly enter the bloodstream [31], [32]. During the initial infection stage, resident cells in the dermis (such as fibroblasts [32], keratinocytes [33], melanocytes, and endothelial cells) are infected and produce viral progeny.

Most infected individuals develop symptoms 3–7 days (range: 1–12 days) after the bite. The clinical presentation is typically characterized by sudden onset of high fever and severe joint pain, which often affects small joints (e.g., wrists and ankles) though larger joints (knees, shoulders) may also be involved. The arthralgia can be so intense that it limits mobility. Other common symptoms include rash, muscle pain, headache, fatigue, and nausea. In rare cases, complications may arise involving the eyes, heart, or nervous system [34].

The initial infection begins in the skin, where CHIKV binds to, infects, or is internalized by antigen-presenting cells. These cells, or free viral particles, then drain via the lymphatic vessels to lymph nodes and into the bloodstream. Once in the circulation, the virus disseminates systemically either as free particles or within infected migratory immune cells, establishing a widespread infection. From the bloodstream, CHIKV spreads throughout the body to secondary sites, such as the muscles, joints, and skin, where it replicates and causes the primary symptoms of the disease.

### 4.1 Acute phase of chikungunya virus infection

While most arboviruses exhibit comparable infection pathways and broad cellular tropism, CHIKV is distinguished by key virological and clinical features. The acute phase of CHIKV infection is characterized by a clinical triad of fever, joint pain (arthralgia), and rash. Patients commonly report additional symptoms including headache, significant fatigue, and muscle pain (myalgia), which collectively contribute to a debilitating and distressing illness [34], [35].

Its hallmark characteristics include a notably short incubation period (often ≤3 days) and exceptionally high viremia. Acting as an amplifying host, humans develop this high-level viremia, which is essential for infecting mosquitoes during a blood meal and sustaining the transmission cycle. Viral replication in the liver and spleen, as well as infection of peripheral endothelial cells and monocytes in the blood, are believed to contribute to the significant high viremia. Clinically, following the short incubation, patients typically present with an abrupt onset of high fever (≥39 °C) that lasts 4–5 days. The viremic phase generally resolves within 8 days after symptoms appear, although it can persist for up to 10–12 days in some cases, correlating with the period of mosquito infectivity.

Joint pain is the hallmark feature of CHIKV infection, typically emerging 2–5 days after the onset of fever. This prominent and often debilitating symptom is usually bilateral and symmetrical, predominantly affecting distal joints such as those in the hands, wrists, feet, and ankles more frequently than proximal joints [36].

Approximately two-thirds of patients with CHIKV infection develop a transient rash during the acute phase, typically presenting as maculopapular eruptions. A cross-sectional study also found that 21% of patients exhibited central facial rash, resembling the malar rash associated with systemic lupus erythematosus [36]. CHIKV's tropism for skin-resident cells is thought to be closely associated with the maculopapular rash commonly observed after infection.

Beyond the classic triad of fever, arthralgia, and rash, CHIKV infection can lead to a range of less common but clinically significant symptoms. Among these, neurological manifestations are particularly severe and are associated with higher rates of ICU admission and mortality. Although rare, CHIKV can invade the central nervous system, causing conditions such as encephalitis, meningoencephalitis, acute disseminated encephalomyelitis, Guillain–Barré syndrome, optic neuropathy, and neuroretinitis. These complications occur more frequently in infants and the elderly and typically present 1–3 weeks after infection [37].

While CHIKV is generally not considered a fatal infection, deaths do occur, primarily in patients with underlying comorbidities or severe neurological involvement, which represents the leading cause of CHIKV-related mortality.

### 4.2 Chronic phase of chikungunya virus infection

While the majority of patients recover fully from the acute phase of CHIKV infection, a significant proportion—estimated between 40% and 80%—progress to a chronic stage characterized by persistent joint pain and functional impairment, with the transition influenced by older age, viral genotype, initial viral load, and pre-existing osteoarthritis [38]. However, the underlying mechanism of chronic arthralgia remains a subject of critical debate, with two competing but not mutually exclusive hypotheses dominating current research. The persistent viral components hypothesis posits that residual CHIKV RNA (but not replication-competent virus) detected in synovial macrophages, fibroblasts, and myofibres of chronic patients sustains low-grade inflammation via continuous activation of pattern recognition receptors (e.g., TLR3) and secretion of pro-inflammatory cytokines (IL-6, GM-CSF) as well as matrix metalloproteinases (MMPs); this is supported by animal models showing that viral RNA persistence correlates with joint pathology and that targeting viral replication intermediates reduces chronic symptoms. In contrast, the autoimmune hypothesis suggests CHIKV infection triggers molecular mimicry or bystander activation of autoreactive immune cells, as evidenced by cross-sectional studies identifying autoantibodies against joint-specific antigens (e.g., collagen II, aggrecan) and expanded T cell clones reactive to self-epitopes in chronic patients—synovial fluid from these individuals lacks detectable viral RNA but exhibits elevated levels of autoantibodies and Th17 cells, which drive joint inflammation in rheumatoid arthritis. Critically, these hypotheses are not mutually exclusive: residual viral components may initiate autoimmune responses that persist independently of viral RNA. The lack of consensus on this mechanism has directly hindered the development of targeted therapeutics—current treatments (e.g., DMARDs) are repurposed from rheumatoid arthritis and lack specificity for CHIKV-induced chronic arthralgia. Resolving this debate requires longitudinal cohort studies combining viral RNA detection, autoantibody profiling, and single-cell sequencing of synovial immune cells to clarify the temporal relationship between viral persistence and autoimmune activation [39].

### 4.3 Neurological manifestations of chikungunya virus infection

CHIKV infection exhibits a spectrum of neurological complications that have become increasingly prominent in recent severe cases, with up to 60% of severe CHIKV cases involving CNS manifestations. These complications span acute to chronic stages and vary by age group, with higher severity observed in infants, the elderly, and immunocompromised individuals.

Acute neurological manifestations primarily include encephalitis, optic neuritis, myeloradiculitis, and Guillain-Barré syndrome (GBS) [40]. Encephalitis, a life-threatening complication, is characterized by altered consciousness, seizures, and focal neurological deficits, with autopsy findings revealing perivascular infiltration of mononuclear cells in the brain parenchyma. Optic neuritis presents with sudden visual impairment, while myeloradiculitis manifests as limb weakness and sensory disturbances due to spinal cord and nerve root inflammation. GBS, an autoimmune-mediated polyneuropathy, occurs as a post-infectious complication, leading to symmetric flaccid paralysis and respiratory compromise in severe cases [41].

Chronic neurological sequelae are equally notable, particularly in elderly patients and children exposed perinatally. Approximately 70% of CHIKV-infected older adults report long-term cognitive impairments, including memory loss and executive dysfunction, which may increase the risk of dementia. Children infected during the perinatal period often develop neurodevelopmental delays by 2 years of age, including deficits in language, motor coordination, socialization, and posture. Additionally, neuropsychiatric complications such as depression and mood disorders are common, linked to persistent inflammatory cytokines (e.g., IL-6, IL-8) and chronic pain [42].

The underlying mechanisms of CNS invasion include two primary pathways: direct viral entry through the choroid plexus and translocation of infected peripheral blood mononuclear cells (PBMCs) across the compromised blood-brain barrier (BBB). CHIKV-induced elevation of chemokine CCL-2 and adhesion molecules (e.g., PECAM-1, ZO-1) increases BBB permeability, facilitating viral dissemination and neuroinflammation [8]. Activated microglia and astrocytes in the CNS release pro-inflammatory cytokines (IL-1β, IL-6, TNF-α) and reactive oxygen species (ROS), leading to synaptic dysfunction, neuronal damage, and neurodegeneration [43].

### 4.4 Neonatal vertical transmission of chikungunya virus infection

Vertical transmission of CHIKV from mother to neonate has emerged as a critical severe manifestation, with distinct clinical outcomes depending on the timing of maternal infection. Transmission primarily occurs when the mother is viremic during labor or within 15 days pre-delivery, with reported transmission rates up to 50% in viremic mothers [44].

Neonatal manifestations are often severe and distinct from adult cases. Newborns exposed perinatally frequently present with sepsis-like syndrome, encephalitis, and neurological abnormalities. Encephalopathy is a major complication, affecting 68.7% of neonates born to infected mothers, characterized by lethargy, seizures, and poor feeding. Fetal distress occurs in 19.6% of cases, and long-term neurodevelopmental sequelae are common, including cognitive and motor deficits [45]. Earlier studies suggested no congenital malformations, but recent evidence indicates that maternal CHIKV infection during pregnancy may be associated with fetal malformations.

Maternal CHIKV infection during late pregnancy carries the highest risk of severe neonatal outcomes, while infection in early pregnancy is linked to fetal loss (2%) and congenital anomalies. The virus can cross the placental barrier, with CHIKV RNA detected in amniotic fluid and fetal tissues in cases of stillbirth [44]. This highlights the need for targeted monitoring of pregnant women in endemic areas and close follow-up of neonates born to infected mothers.

## 5 Chikungunya virus associated immunopathology

The pathogenesis of chikungunya disease (CHIKVD) involves a complex interplay of viral factors, host immune responses, and interactions with specific cellular pathways. A particular challenge lies in the dual role of the host immune response: while essential for clearing the infection, it also contributes significantly to tissue damage and disease manifestations. In the acute phase, the virus further infects macrophages and fibroblasts in the synovial joints, triggering tissue destruction, promoting the release of pro-inflammatory cytokines, and attracting immune cell infiltration (including macrophages, T cells, B cells, and natural killer cells). This creates a sustained inflammatory environment, ultimately resulting in the characteristic arthritis-like joint pain.

During the acute phase of arthritogenic CHIKV infection, monocytes, macrophages, and type I interferons (e.g., IFN-α/β) play a critical protective role by suppressing viral replication [46]. Consistent with this role, clinical analyses reveal that disease severity is associated with distinct immune signatures, notably elevated MCP-1/CCL2 and IL-6 [47], alongside reduced IL-8 [48]. Furthermore, CHIKV can directly infect human osteoblasts, upregulating interleukin-6 (IL-6)—a cytokine linked to both disease severity and chronicity [49]. Studies on chronic CHIKV disease have further linked persistent arthralgia to a broader profile of elevated inflammatory mediators, including IL-1β, IL-6, IL-8, MCP-1/CCL2, MMP-1, MMP-3, and GM-CSF [50]. Among these, IL-6 emerges as a promising candidate biomarker for both severe acute disease and chronic progression—clinical analyses show IL-6 levels correlate with acute fever severity and long-term joint pain duration. Additionally, exhausted CD8+ T cells (associated with failed viral clearance) and autoantibodies targeting joint-specific antigens (collagen II, aggrecan) have been detected in chronic patients, suggesting their potential as prognostic markers for chronic arthralgia. However, validation of these candidates requires large-scale prospective cohort studies tracking patients from acute infection to chronic phase, with serial sampling of cytokines, T-cell subsets, and autoantibodies to establish temporal associations with disease progression.

Following this innate immune response, the adaptive immunity against CHIKV consists of two essential branches: cellular immunity, mediated by T and B cells, and humoral immunity, driven by antibodies. Both are critical for controlling infection. The cellular immune response to CHIKV involves the coordinated activation of multiple immune cell types. Among these, CD4+ T cells are one of the most extensively studied and have been shown to play a key role in driving inflammation during CHIKVD [51]. Research has demonstrated that TCR-deficient mice infected with CHIKV develop joint inflammation only after receiving splenic CD4+ T cells from infected wild-type mice, underscoring the central role of these cells in mediating joint damage [52], [53]. Despite their pathogenic contribution, CD4+ T cells remain essential for the formation of immunological memory, which is critical for long-term antiviral adaptive immunity. A robust CD8+ T cell response is triggered during acute CHIKV infection, leading to the elimination of virus-infected cells. Elevated early viral titers are associated with the development of exhausted CD8+ T cells and a corresponding reduction in their capacity to clear infected cells. Studies using CD8+ deficient mice show that these cells do not mitigate joint pathology, suggesting they provide little protective effect against CHIKV-induced damage.

In the initial phase of CHIKV infection, B cells generate virus-specific IgM antibodies [54]. IgM antibodies preferentially bind to epitopes on the CHIKV E1 and E2 glycoproteins. Their early neutralization, detectable around day 6, is associated with reduced viraemia, while later control of infection is predominantly mediated by IgG antibodies [55].

**Fig. 4:**
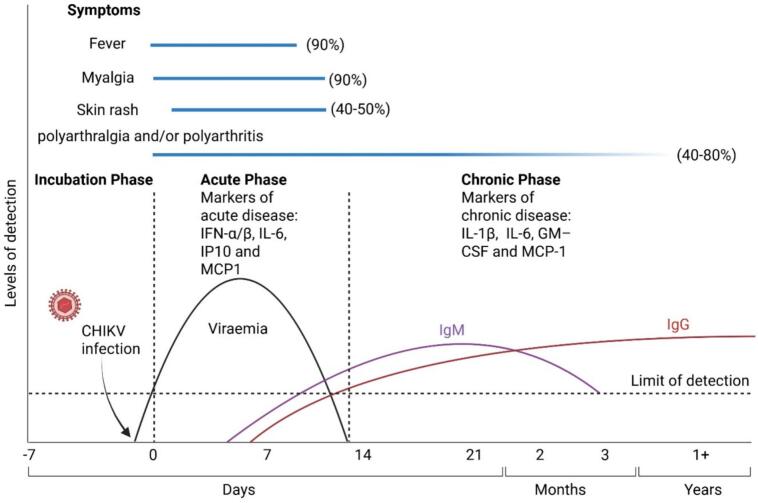
CHIKV-associated immunopathology. Schematic depiction of the disease course divided into three phases: incubation, acute, and chronic. Following infection, the acute phase is characterized by viremia and systemic inflammation driven by mediators such as IFN-α/β, IL-6, IP-10, and MCP-1. Viremia typically resolves within two weeks, marking the transition. A subset of patients progresses to the chronic phase, which is defined by persistent musculoskeletal symptoms and elevated markers including IL-1 β, IL-6, GM-CSF and MCP-1.

Activation of B cells by CHIKV antigens via the B cell receptor (BCR) [56], along with cytokine and co-stimulatory signals, induces their proliferation and differentiation into antibody-secreting plasma cells. These antibodies are primarily directed against CHIKV envelope proteins (Fig. 4).

## 6 Diagnosis and screening of chikungunya virus infection

The accurate and timely diagnosis of CHIKV infection is vital for effective patient management and robust public health control. For patients presenting with fever and polyarthralgia—especially travelers returning from endemic areas—applying specific diagnostic tests is crucial to confirm infection and guide therapeutic decisions. Beyond individual care, timely diagnosis is fundamental to epidemiological surveillance. Confirming cases in sentinel populations generates critical data that facilitates early outbreak detection and informs targeted interventions.

Clinical evaluation for suspected CHIKV infection typically starts with an assessment of symptoms—most commonly sudden fever, arthralgia, rash, and other influenza-like manifestations. Although these clinical features are non-specific and overlap considerably with other arboviral diseases such as dengue and Zika virus infection, they nevertheless offer valuable initial diagnostic clues.

Given the non-specific nature of these clinical features, laboratory confirmation is essential to differentiate CHIKV infection from other arboviral diseases. Diagnostic methods include virus isolation, viral nucleic acid detection (RT-PCR), and virus-specific antibody testing. Among these, detection of CHIKV RNA in serum or successful virus isolation provides definitive confirmation of infection.

### 6.1 Viral nucleic acid detection

The gold standard for diagnosing acute CHIKV infection is reverse-transcription polymerase chain reaction (RT-PCR), which detects viral RNA in blood, serum, or plasma with high sensitivity and specificity. This method provides conclusive evidence of active infection, particularly within the first 8 days after symptom onset, and has been widely validated in numerous studies for its strong diagnostic performance [57].

Nucleic acid testing is indispensable for the diagnosis of CHIKV infection. Beyond the laboratory, these assays can be deployed in non-laboratory settings using portable, compact devices capable of operation via mains electricity or battery power. Equipped with data transmission capabilities, such systems facilitate real-time monitoring and support rapid public health response during outbreaks [58].

### 6.2 Antibody detection

When direct viral detection is not feasible, serological methods provide an alternative means of diagnosis. This can be achieved through the identification of CHIKV-specific IgM and IgG antibodies via paired serology, or by demonstrating a fourfold or greater increase in antibody titers between acute- and convalescent-phase serum samples—typically collected 7 to 14 days apart. The enzyme-linked immunosorbent assay (ELISA) is the most widely used platform for detecting anti-CHIKV IgM and IgG antibodies in serum or plasma.

CHIKV-specific IgM antibodies typically become detectable near the end of the first week of illness (around 4–7 days after symptom onset), peak between 3 and 5 weeks and often remain detectable for approximately two months. However, IgM serology should be interpreted with caution, as it may yield false-positive results due to cross-reactivity with other alphaviruses and cannot provide definitive diagnostic evidence alone. For patients with negative results during the acute phase, it is recommended to collect a convalescent-phase serum sample 7–14 days after the first sample. Confirmation of CHIKV infection is established through IgG seroconversion or a ≥ 4-fold rise in IgG antibody titers between the acute and convalescent samples [59].

## 7 Antiviral therapeutic strategies against chikungunya virus

The development of safe and effective vaccines and antivirals is essential for global control of CHIKV infection. A robust humoral immune response is strongly associated with viral clearance and sustained protection, underscoring the importance of vaccine development. In parallel, numerous antiviral candidates are currently under investigation to expand the available therapeutic options [60].

### 7.1 Chikungunya virus vaccine candidates

The development of a safe and effective CHIKV vaccine is essential for global control of the virus, given that a strong antibody-mediated immune response is critical for clearing the infection and providing protective immunity. Multiple CHIKV vaccine candidates have been advanced through various stages of preclinical and clinical evaluation. The CHIKV vaccine candidates are presented in Table 2.

**Table 2:** Chikungunya virus vaccine candidates.

Vaccine	Type	Advantages	Limitations	Status
VLA1553	Live-attenuated virus	Safe and well-tolerated Single-dose Rapid immune response	May cause transient arthralgia and fever Contraindicated in pregnancy and immunocompromised individuals Limited clinical data across age and ethnic groups Currently higher cost	Licensed in the U.S., Canada, and E.U. Phase III completed; FDA application submitted August 2022
PXVX0317	Virus-like particle	Safe and well-tolerated Single-dose Rapid response Durable immunity Suitable for high-risk groups	Limited clinical data across diverse populations Protection against chronic disease remains uncertain Adjuvant-dependent	Licensed in the U.S. and E.U. with Phase III trials ongoing
MV-CHIK/V184	Measles vaccine	Safe and well-tolerated Enhances measles immunity Lack of nsPs may improve safety profile	May require a two-dose regimen Long-term durability remains unknown Effectiveness against diverse CHIKV strains not yet established Limited clinical data across age and ethnic groups	Phase III completed; halted Feb 2023
BBV87	Inactivated virus	Thermostable Safe for pregnancy and immunocompromised individuals Non-replicating design improves safety	Requires two-dose regimen and an adjuvant Efficacy against diverse CHIKV strains remains uncertain Limited clinical data across age and ethnic groups	Phase II/III ongoing
mRNA-1388	mRNA vaccine	Safe and well-tolerated Durable immunity	Requires multiple doses for optimal response Efficacy against diverse CHIKV strains unconfirmed Limited data across age and ethnic groups Protection against chronic disease not established	Phase I complete

VLA1553, a single-dose live-attenuated vaccine, represents the most advanced CHIKV vaccine candidate and was approved in the United States and European Union in 2023, followed by Canada in 2024. Its design features a 60-amino acid deletion in the hypervariable domain of nsP3 [61], [62]. The vaccine has demonstrated high tolerability in low- and medium-dose groups over multiple years of research, while also eliciting robust immunogenicity and high seroconversion rates [63]. VLA1553 is an FDA-approved vaccine, commercially available in the U.S. since late 2023 for adults at elevated risk of CHIKV exposure [64]. It met all primary and secondary endpoints in a Phase III placebo-controlled trial, though it may cause prolonged chikungunya-like side effects. Marketed in the U.S. under the trade name IXCHIQ, this vaccine marked the first FDA-approved prophylaxis for CHIKV, addressing a critical unmet public health need. However, post-authorization implementation and surveillance during 2024–2025 revealed nuanced real-world challenges that refined its clinical application. Specifically, while the pivotal Phase III trial demonstrated favorable safety in 3082 participants (including 346 adults ≥65 years), subsequent real-world data from the CDC and FDA identified emerging safety signals, particularly an increased risk of severe adverse events (SAEs), including neurological complications, in the elderly population. These findings prompted significant regulatory adjustments across regions in 2025: the EU and UK issued age restrictions advising against use in adults ≥65 years, while the U.S. temporarily suspended its use pending further review. This implementation experience highlights the critical importance of post-approval pharmacovigilance for live-attenuated vaccines, especially when deployed in demographically diverse populations amid ongoing global CHIKV outbreaks [65].

PXVX0317 is a virus-like particle (VLP)-based vaccine adjuvanted with aluminum hydroxide [66]. It has been evaluated in Phase II clinical trials, where it demonstrated strong immunogenicity by inducing neutralizing antibodies and activating antigen-specific B cells in human participants [67]. A single high-dose regimen of PXVX0317 is now advancing to Phase III trials and has received Fast Track designation from the U.S. Food and Drug Administration (FDA).

Beyond live-attenuated and VLP vaccines, another approach uses viral vectors to deliver CHIKV antigens. The frontrunner is V184 (MV-CHIK), a vaccine made from a weakened measles virus (Schwarz strain). This viral vector has been engineered to carry the structural genes of the CHIKV, specifically from the 2006 La Réunion Island strain [68], [69].

Like VLP vaccines, mRNA vaccines are safer than live-attenuated vaccines for pregnant and immunocompromised individuals. Separately, inactivated virus vaccines have also been developed to prevent CHIKV. BBV87 is an inactivated whole-virion chikungunya vaccine candidate developed by Bharat Biotech. It is derived from the DRDE-06 strain, which was isolated during the 2006 Indian outbreak of the ECSA genotype, and is manufactured using Vero cell culture. The virus is rendered non-infectious through beta-propiolactone inactivation. A Phase-I clinical trial conducted in healthy adults has evaluated the safety, tolerability, and immunogenicity of this vaccine candidate [70].

The mRNA-based vaccine candidate mRNA-1388 (VAL-181388), developed by Moderna, encodes the full structural polyprotein of a West African chikungunya virus genotype. This includes the capsid and the envelope proteins (E3, E2, 6 k/TF, and E1). The mRNA sequence is delivered via a lipid nanoparticle (LNP) delivery system to facilitate cellular uptake and protein expression [71]. The Phase I trial demonstrated that mRNA-1388 was safe and well-tolerated. It elicited strong, durable humoral immune responses characterized by dose-dependent increases in neutralizing antibody titers, which remained elevated for up to one year compared to the placebo group [71].

A key unmet need and knowledge limitation remains the lack of robust clinical data on vaccine safety and efficacy in vulnerable populations, including infants, the elderly, pregnant women, and immunocompromised individuals, which restricts the broad clinical application of currently licensed vaccines.

### 7.2 Integrated strategies for chikungunya virus control and surveillance

Since CHIKV is transmitted mainly by *Aedes aegypti* and *Aedes albopictus* mosquitoes, controlling these vectors is essential for reducing infection rates. Surveillance of mosquito populations in high-risk areas is a key strategy, as it enables early warning of potential outbreaks and guides targeted public health responses [72], [73]. Vector control strategies aim to reduce the population of disease-carrying mosquitoes, ultimately diminishing the risk of viral transmission to humans.

Currently, the primary approach to controlling the transmission of arthritogenic alphaviruses still relies on mosquito vector management and reducing human-mosquito contact. This strategy integrates disease surveillance with entomological monitoring while implementing comprehensive interventions targeting all life cycle stages of mosquitoes in and around residential areas.

Traditional mosquito control measures, which can theoretically be employed to curb outbreaks of chikungunya fever, mainly include chemical interventions—such as indoor residual spraying, space spraying, fogging, insecticide-treated nets, and larvicides—as well as biological and mechanical methods like the introduction of larvivorous fish, environmental modification, and the elimination of stagnant water sources to reduce mosquito breeding sites [74]. However, in practice, the effectiveness of these conventional strategies is gradually declining. This is due to several challenges: the widespread development of mosquito resistance to insecticides; behavioral adaptations in vectors, such as a shift toward outdoor biting and altered resting habits (e.g., *Aedes aegypti*'s indoor resting behavior limits the impact of outdoor spraying); human population movement; and the expansion of mosquito habitats into new areas driven by climate change. Furthermore, CHIKV has fully adapted to urban transmission cycles, leading to outbreaks increasingly in densely populated urban environments where large-scale use of toxic chemical sprays is less feasible and environmentally concerning. As a result, while these traditional methods remain part of integrated vector management approaches, their long-term utility is constrained by rising costs, operational limitations, and growing ecological considerations.

To address the complex transmission dynamics of CHIKV—characterized by interactions between human hosts, mosquito vectors, and environmental drivers—a robust “One Health” surveillance and early warning system is urgently required. This architecture integrates three core modules with cross-sectoral collaboration: Human case surveillance serves as the “signal core” by expanding detection coverage (combining RT-PCR for acute cases and serological testing for retrospective diagnosis in endemic/border/tourist areas), monitoring special populations (neonates, the elderly, and patients with comorbidities) to track severe outcomes, and linking case data with GIS for transmission hotspot mapping. Mosquito vector surveillance bridges human cases and the environment through standardized entomological monitoring (ovitraps, BG-Sentinel traps) to track Aedes density (Breteau Index, Container Index), viral carriage/genotype analysis (RT-PCR and sequencing to identify circulating lineages and adaptive mutations), and insecticide resistance testing to guide vector control. Environmental surveillance identifies pre-outbreak signals by real-time tracking of climatic factors (temperature, rainfall) for vector proliferation forecasting, mapping anthropogenic/ecological drivers (urbanization, deforestation, extreme events), and monitoring non-human primates (NHPs) in forested areas to warn of sylvatic-to-human spillover risks.

The system's effectiveness relies on One Health integration: establishing a unified data platform to synergize human, mosquito, environmental, and NHP monitoring data across health, entomological, meteorological, and veterinary departments; developing multi-factor predictive models that combine case incidence, vector density, viral carriage rate, and environmental suitability to set threshold triggers (e.g., BI >50, CHIKV-positive mosquitoes detected) for tiered responses (public health alerts, targeted insecticide spraying); and forming cross-sectoral task forces for joint investigations and integrated interventions. This streamlined framework enables early warning and rapid response, addressing the interconnectedness of human, animal, and environmental health in CHIKV transmission.

The development of genetic control technologies aims to suppress or genetically modify mosquito populations, consequently interrupting the transmission cycle of mosquito-borne viral pathogens. Successful vector control interventions include the field release of male mosquitoes infected with the intracellular bacterium *Wolbachia*, which suppresses wild *Aedes aegypti* populations and curbs the transmission of CHIKV and other arboviruses [75], [76], [77], as well as the deployment of transgenic mosquitoes engineered to produce sterile females or inhibit viral replication of target pathogens.

The expanding geographic range of mosquito vectors due to climate change and increasing global travel underscores the growing importance of robust disease surveillance. Monitoring trends in transmission and prevalence is vital for anticipating and responding to shifts in disease distribution. Furthermore, integrating regional epidemiological data into clinical decision-support systems can significantly improve the accuracy of differential diagnosis for acute febrile illnesses. This data-driven approach also facilitates the identification of optimal locations for clinical trials of vaccines and therapeutics, ensuring that research efforts are focused in high-prevalence areas.

While no single mosquito control measure is likely to achieve complete effectiveness against CHIKV on its own, integrating these approaches into a coordinated strategy—combined with synergistic vector control methods and vaccines—holds promise for effectively controlling CHIKV transmission.

### 7.3 Current treatment of chikungunya virus

Currently, no licensed antivirals or specific therapeutics are available for the treatment of arthritogenic alphavirus infections, including CHIKV. As a result, clinical management remains primarily supportive and focuses on symptomatic relief. This includes the use of analgesics, antipyretics, nonsteroidal anti-inflammatory drugs, hydration, and psychological support. Nonetheless, several investigational compounds have demonstrated promising antiviral activity against CHIKV both in vitro and in vivo, through mechanisms such as inhibiting nsPs or disrupting viral replication.

Aspirin is contraindicated in the acute phase because the clinical presentation of CHIKV can mimic that of dengue fever. Its well-known anticoagulant properties could precipitate or worsen hemorrhagic complications, such as those seen in dengue hemorrhagic fever.

Disease-modifying antirheumatic drugs (DMARDs), such as hydroxychloroquine and methotrexate, function as immunomodulatory and anti-inflammatory agents through pleiotropic mechanisms. They have been utilized in the treatment of chronic chikungunya fever, where they demonstrate efficacy in controlling joint symptoms by targeting chronic pro-inflammatory processes and disrupting key inflammatory pathways. However, the use of DMARDs is generally reserved for severe chronic cases and is cautioned against during acute infection due to the risk of exacerbating symptoms. Methotrexate is the first-line therapeutic choice, particularly for chronic inflammatory rheumatic diseases, including rheumatoid arthritis-like presentations that may occur in severe clinical cases of chronic chikungunya. Its use should be guided by clinical response and tolerability, with appropriate monitoring for potential adverse effects.

Notably, the absence of specific antiviral therapeutics for CHIKV remains a critical knowledge gap, and the short viremic window of acute infection further narrows the time window for potential antiviral intervention, posing major challenges for clinical treatment of both acute and chronic cases.

## 8 Conclusions

CHIKV remains a substantial global public health threat, characterized by its propensity to cause large-scale outbreaks and the significant long-term morbidity associated with infection. However, to date, no monoclonal antibodies targeting alphaviruses have been approved for human treatment or prevention. Although animal studies have demonstrated their feasibility, practical applications remain limited due to high costs and complexities in administration.

In the absence of effective vaccines, antiviral therapies against CHIKV may help reduce viremia during the acute phase, potentially limiting progression to chronic disease. However, similar to other arboviral infections, CHIKV symptoms often emerge abruptly, and the viremic window is short, leaving a narrow time frame for intervention after diagnosis.

Therefore, developing highly safe antiviral agents is critical not only for acute treatment but also for prophylactic use—such as in travelers to endemic areas or household contacts of infected individuals.

CHIKV has become a major public health burden and global threat. Therefore, multiple measures are needed to mitigate the disease burden caused by CHIKV while preparing for potential threats from other arthritogenic alphaviruses (including CHIKV re-emergence).

First, strengthening molecular epidemiological surveillance, rapid diagnosis, and timely treatment is crucial for interrupting transmission chains, improving patient outcomes, and formulating effective public health policies. Second, providing accessible vaccines for high-risk populations is key to controlling CHIKV transmission and reducing its disease burden. Priority should be given to developing broad-spectrum vaccines targeting multiple arthritogenic alphaviruses—given that some vaccines can induce long-lasting immunity, the elimination of CHIKV from urban transmission cycles may be achievable.

Third, developing highly effective, safe, and accessible antiviral drugs and monoclonal antibody therapies is essential for treating severe and chronic cases. Achieving this goal also requires optimizing or establishing novel experimental models that accurately replicate human disease characteristics. Fourth, identifying biomarkers associated with severe and chronic disease progression will enhance clinical management and reduce disease burden. Finally, a deeper understanding of the spatiotemporal transmission patterns of arthritogenic alphaviruses in urban and forest environments, as well as the impact of climate change on vectors (including human hosts), is critical for formulating effective mitigation strategies.

## 9 Future directions and unmet research needs

From a One Health perspective, addressing CHIKV burden requires integrating insights from human clinical research, animal ecology, and environmental vector management. Building on the current advances summarized herein, several critical research gaps and future directions merit prioritization:

Despite significant advances in CHIKV research, several critical knowledge gaps remain that hinder the development of optimal interventions. Future research should focus on elucidating the precise cellular and molecular mechanisms underlying chronic arthralgia, including the role of persistent viral RNA, synovial fibroblast activation, and inflammatory pathways such as IL-6 and MMPs. Additionally, the potential association between viral genotypes (ECSA, Asian, West African) and clinical outcomes, including disease severity, chronicity risk, and drug resistance, warrants further epidemiological and experimental investigation.

To address clinical challenges, prospective cohort studies are needed to identify reliable biomarkers (e.g., specific cytokines, T-cell subsets, autoantibodies) that can predict severe disease and chronic progression. These studies should enroll patients within 72 h of acute symptom onset, with longitudinal follow-up (12–24 months) to track inflammatory cytokines (IL-6, MMP-3) as predictors of acute severity, T-cell subset dynamics (exhausted CD8+ T cells, Th17 cells) linked to chronic inflammation, and persistent autoantibodies (anti-collagen II, anti-aggrecan) associated with joint pathology. Integrating these markers into a composite prognostic panel would enable early identification of high-risk patients for targeted interventions. The development and validation of novel animal models, particularly non-human primate systems that recapitulate human joint pathology, will be crucial for testing new therapeutics—aligning with One Health's focus on cross-species translational research.

On the public health front, translating research into policy is imperative. This includes conducting field trials to evaluate the efficacy of integrated vector management (IVM) strategies combining Wolbachia-based control with traditional methods in high-density urban areas. Furthermore, implementation studies focusing on enhancing molecular epidemiological surveillance, developing rapid point-of-care diagnostics, and establishing timely treatment protocols are essential to interrupt transmission chains and improve patient care in resource-limited endemic regions. These critical knowledge limitations—including the lack of specific antivirals, the narrow viremic window for intervention, and insufficient vaccine data in vulnerable groups—underscore the urgent need for targeted future research to address these unmet public health needs.

## CRediT authorship contribution statement

**Shiqin Dai:** Writing – review & editing. **Hao Li:** Writing – review & editing. **Shu Li:** Writing – review & editing. **Xinhua Shao:** Writing – review & editing. **Xianliang Cheng:** Writing – review & editing.

## Consent for publication

Written informed consent for publication was obtained from all participants.

## Ethics approval and consent to participate

Not applicable. This study is a review/article based on existing published literature and does not involve direct human or animal subjects.

## Declaration of generative AI and AI-assisted technologies in the writing process

During the preparation of this work the authors used DeepSeek in order to improve readability and language. After using this tool/service, the authors reviewed and edited the content as needed and take full responsibility for the content of the published article.

## Funding

Not applicable.

## Declaration of competing interest

The authors declare that they have no competing interests.

## Data Availability

Not applicable.

## References

[1] Brito C., Falcao M.B., de Albuquerque M., Cerqueira-Silva T., Teixeira M.G., Franca R.F.O. (2025). Chikungunya: from hypothesis to evidence of increased severe disease and fatalities. Viruses.

[2] Yang Y.F., Qiu Y.B., Xu Q., Gao R.C., Tang T., Tian Y. (2025). Mapping the global risk of chikungunya virus endemicity and autochthonous transmission following importation. Travel Med. Infect. Dis..

[3] Ribeiro Dos Santos G., Jawed F., Mukandavire C., Deol A., Scarponi D., Mboera L.E.G. (2025). Global burden of chikungunya virus infections and the potential benefit of vaccination campaigns. Nat. Med..

[4] Iwamura T., Guzman-Holst A., Murray K.A. (2020). Accelerating invasion potential of disease vector *Aedes aegypti* under climate change. Nat. Commun..

[5] Kraemer M.U.G., Reiner R.C., Brady O.J., Messina J.P., Gilbert M., Pigott D.M. (2019). Publisher correction: past and future spread of the arbovirus vectors *Aedes aegypti* and *Aedes albopictus*. Nat. Microbiol..

[6] Bettis A.A., L’Azou Jackson M., Yoon I.K., Breugelmans J.G., Goios A., Gubler D.J. (2022). The global epidemiology of chikungunya from 1999 to 2020: a systematic literature review to inform the development and introduction of vaccines. PLoS Negl. Trop. Dis..

[7] Rama K., de Roo A.M., Louwsma T., Hofstra H.S., Gurgel do Amaral G.S., Vondeling G.T. (2024). Clinical outcomes of chikungunya: a systematic literature review and meta-analysis. PLoS Negl. Trop. Dis..

[8] de Souza W.M., Fumagalli M.J., de Lima S.T.S., Parise P.L., Carvalho D.C.M., Hernandez C. (2024). Pathophysiology of chikungunya virus infection associated with fatal outcomes. Cell Host Microbe.

[9] Giovanetti M., Vazquez C., Lima M., Castro E., Rojas A., Gomez de la Fuente A. (2023). Rapid epidemic expansion of chikungunya virus east/central/South African lineage, Paraguay. Emerg. Infect. Dis..

[10] Wan S., Zhang X., Cong X., Liu Y., Huang S., Zhou M. (2025). Viral load dynamics of chikungunya virus in human specimens - Foshan city, Guangdong province, China, 2025. China CDC Wkly..

[11] Wang T.Y., Sun Y., Tang Y.D. (2025). Re-emergence of chikungunya virus in China by 2025: what we know and what to do?. PLoS Pathog..

[12] Zhang Y., Zhuo Z., Huang Y., Zhong X., Mo X., Li L. (2025). Genomic characterization and evolutionary analysis of chikungunya virus strains in Guangzhou, China, 2025. Virol. J..

[13] He F., Liang Y., Gong Y., Li P., Yu J., Wei C. (2026). Clinical epidemiology and viral genomics insights from a chikungunya fever outbreak in South China, 2025. Front. Cell. Infect. Microbiol..

[14] Sharif N., Sarkar M.K., Ferdous R.N., Ahmed S.N., Billah M.B., Talukder A.A. (2021). Molecular epidemiology, evolution and reemergence of chikungunya virus in South Asia. Front. Microbiol..

[15] de Souza W.M., Lecuit M., Weaver S.C. (2025). Chikungunya virus and other emerging arthritogenic alphaviruses. Nat. Rev. Microbiol..

[16] Button J.M., Qazi S.A., Wang J.C., Mukhopadhyay S. (2020). Revisiting an old friend: new findings in alphavirus structure and assembly. Curr. Opin. Virol..

[17] Frolov I., Frolova E.I. (2022). Molecular virology of chikungunya virus. Curr. Top. Microbiol. Immunol..

[18] Basore K., Kim A.S., Nelson C.A., Zhang R., Smith B.K., Uranga C. (2019). Cryo-EM structure of chikungunya virus in complex with the Mxra8 receptor. Cell.

[19] Zhang R., Kim A.S., Fox J.M., Nair S., Basore K., Klimstra W.B. (2018). Mxra8 is a receptor for multiple arthritogenic alphaviruses. Nature.

[20] Prado Acosta M., Geoghegan E.M., Lepenies B., Ruzal S., Kielian M., Martinez M.G. (2019). Surface (s) layer proteins of *Lactobacillus acidophilus* block virus infection via DC-SIGN interaction. Front. Microbiol..

[21] Jones J.E., Long K.M., Whitmore A.C., Sanders W., Thurlow L.R., Brown J.A. (2017). Disruption of the opal stop codon attenuates chikungunya virus-induced arthritis and pathology. mBio.

[22] Zhang K., Law M.C.Y., Nguyen T.M., Tan Y.B., Wirawan M., Law Y.S. (2022). Molecular basis of specific viral RNA recognition and 5′-end capping by the chikungunya virus nsP1. Cell Rep..

[23] Ramsey J., Mukhopadhyay S. (2017). Disentangling the frames, the state of research on the alphavirus 6K and TF proteins. Viruses.

[24] Brown R.S., Anastasakis D.G., Hafner M., Kielian M. (2020). Multiple capsid protein binding sites mediate selective packaging of the alphavirus genomic RNA. Nat. Commun..

[25] Miao X., Law M.C.Y., Kumar J., Chng C.P., Zeng Y., Tan Y.B. (2025). Saddle curvature association of nsP1 facilitates the replication complex assembly of chikungunya virus in cells. Nat. Commun..

[26] Bae S., Lee J.Y., Myoung J. (2020). Chikungunya virus nsP2 impairs MDA5/RIG-I-mediated induction of NF-kappaB promoter activation: a potential target for virus-specific therapeutics. J. Microbiol. Biotechnol..

[27] Tan Y.B., Chmielewski D., Law M.C.Y., Zhang K., He Y., Chen M. (2022). Molecular architecture of the chikungunya virus replication complex. Sci. Adv..

[28] Elmasri Z., Nasal B.L., Jose J. (2021). Alphavirus-induced membrane rearrangements during replication, assembly, and budding. Pathogens.

[29] Rangel M.V., McAllister N., Dancel-Manning K., Noval M.G., Silva L.A., Stapleford K.A. (2022). Emerging chikungunya virus variants at the E1-E1 interglycoprotein spike interface impact virus attachment and inflammation. J. Virol..

[30] Song H., Zhao Z., Chai Y., Jin X., Li C., Yuan F. (2019). Molecular basis of arthritogenic alphavirus receptor MXRA8 binding to chikungunya virus envelope protein. Cell.

[31] Agarwal A., Joshi G., Nagar D.P., Sharma A.K., Sukumaran D., Pant S.C. (2016). Mosquito saliva induced cutaneous events augment chikungunya virus replication and disease progression. Infect. Genet. Evol..

[32] Wichit S., Diop F., Hamel R., Talignani L., Ferraris P., Cornelie S. (2017). *Aedes Aegypti* saliva enhances chikungunya virus replication in human skin fibroblasts via inhibition of the type I interferon signaling pathway. Infect. Genet. Evol..

[33] Zhang X., Huang Y., Wang M., Yang F., Wu C., Huang D. (2018). Differences in genome characters and cell tropisms between two chikungunya isolates of Asian lineage and Indian Ocean lineage. Virol. J..

[34] Mourad O., Makhani L., Chen L.H. (2022). Chikungunya: an emerging public health concern. Curr. Infect. Dis. Rep..

[35] Suhrbier A. (2019). Rheumatic manifestations of chikungunya: emerging concepts and interventions. Nat. Rev. Rheumatol..

[36] Paul B.J., Sadanand S. (2018). Chikungunya infection: a re-emerging epidemic. Rheumatol. Ther..

[37] Cerny T., Schwarz M., Schwarz U., Lemant J., Gerardin P., Keller E. (2017). The range of neurological complications in chikungunya fever. Neurocrit. Care..

[38] Kawai K., Kawai A.T., Wollan P., Yawn B.P. (2017). Adverse impacts of chronic pain on health-related quality of life, work productivity, depression and anxiety in a community-based study. Fam. Pract..

[39] Heath C.J., Lowther J., Noel T.P., Mark-George I., Boothroyd D.B., Mitchell G. (2018). The identification of risk factors for chronic chikungunya arthralgia in Grenada, West Indies: a cross-sectional cohort study. Open Forum Infect. Dis..

[40] Ortiz-Quezada J., Rodriguez E.E., Hesse H., Molina L., Duran C., Lorenzana I. (2021). Chikungunya encephalitis, a case series from an endemic country. J. Neurol. Sci..

[41] Agarwal A., Vibha D., Srivastava A.K., Shukla G., Prasad K. (2017). Guillain-Barre syndrome complicating chikungunya virus infection. J. Neurovirol..

[42] Murillo-Zamora E., Mendoza-Cano O., Trujillo-Hernandez B., Trujillo X., Huerta M., Guzman-Esquivel J. (2018). Screening for depressive mood during acute chikungunya infection in primary healthcare settings. Int. J. Environ. Res. Public Health.

[43] Bersano A., Engele J., Schafer M.K.E. (2023). Neuroinflammation and brain disease. BMC Neurol..

[44] Ferreira F., da Silva A.S.V., Recht J., Guaraldo L., Moreira M.E.L., de Siqueira A.M. (2021). Vertical transmission of chikungunya virus: a systematic review. PLoS One.

[45] van Ewijk R., Huibers M.H.W., Manshande M.E., Ecury-Goossen G.M., Duits A.J., Calis J.C. (2021). Neurologic sequelae of severe chikungunya infection in the first 6 months of life: a prospective cohort study 24-months post-infection. BMC Infect. Dis..

[46] Fox J.M., Roy V., Gunn B.M., Huang L., Edeling M.A., Mack M. (2019). Optimal therapeutic activity of monoclonal antibodies against chikungunya virus requires Fc-FcgammaR interaction on monocytes. Sci. Immunol..

[47] Ng L.F.P. (2017). Immunopathology of chikungunya virus infection: lessons learned from patients and animal models. Annu. Rev. Virol..

[48] Lohachanakul J., Phuklia W., Thannagith M., Thonsakulprasert T., Ubol S. (2012). High concentrations of circulating interleukin-6 and monocyte chemotactic protein-1 with low concentrations of interleukin-8 were associated with severe chikungunya fever during the 2009-2010 outbreak in Thailand. Microbiol. Immunol..

[49] Roy E., Shi W., Duan B., Reid S.P. (2020). Chikungunya virus infection impairs the function of osteogenic cells. mSphere.

[50] Ninla-Aesong P., Mitarnun W., Noipha K. (2019). Proinflammatory cytokines and chemokines as biomarkers of persistent arthralgia and severe disease after chikungunya virus infection: a 5-year follow-up study in southern Thailand. Viral Immunol..

[51] Mapalagamage M., Weiskopf D., Sette A., De Silva A.D. (2022). Current understanding of the role of T cells in chikungunya, dengue and zika infections. Viruses.

[52] Teo T.H., Chan Y.H., Lee W.W., Lum F.M., Amrun S.N., Her Z. (2017). Fingolimod treatment abrogates chikungunya virus-induced arthralgia. Sci. Transl. Med..

[53] Teo T.H., Howland S.W., Claser C., Gun S.Y., Poh C.M., Lee W.W. (2018). Co-infection with chikungunya virus alters trafficking of pathogenic CD8(+) T cells into the brain and prevents plasmodium-induced neuropathology. EMBO Mol. Med..

[54] Chua C.L., Sam I.C., Chiam C.W., Chan Y.F. (2017). The neutralizing role of IgM during early chikungunya virus infection. PLoS One.

[55] Powers J.M., Lyski Z.L., Weber W.C., Denton M., Streblow M.M., Mayo A.T. (2023). Infection with chikungunya virus confers heterotypic cross-neutralizing antibodies and memory B-cells against other arthritogenic alphaviruses predominantly through the B domain of the E2 glycoprotein. PLoS Negl. Trop. Dis..

[56] Lam J.H., Smith F.L., Baumgarth N. (2020). B cell activation and response regulation during viral infections. Viral Immunol..

[57] Natrajan M.S., Rojas A., Waggoner J.J. (2019). Beyond fever and pain: diagnostic methods for chikungunya virus. J. Clin. Microbiol..

[58] Karlikow M., da Silva S.J.R., Guo Y., Cicek S., Krokovsky L., Homme P. (2022). Field validation of the performance of paper-based tests for the detection of the zika and chikungunya viruses in serum samples. Nat. Biomed. Eng..

[59] Bartholomeeusen K., Daniel M., LaBeaud D.A., Gasque P., Peeling R.W., Stephenson K.E. (2023). Chikungunya fever. Nat. Rev. Dis. Primers.

[60] Hucke F.I.L., Bugert J.J. (2020). Current and promising antivirals against chikungunya virus. Front. Public Health.

[61] Meertens L., Hafirassou M.L., Couderc T., Bonnet-Madin L., Kril V., Kummerer B.M. (2019). FHL1 is a major host factor for chikungunya virus infection. Nature.

[62] Ng W.H., Liu X., Ling Z.L., Santos C.N.O., Magalhaes L.S., Kueh A.J. (2023). FHL1 promotes chikungunya and o’nyong-nyong virus infection and pathogenesis with implications for alphavirus vaccine design. Nat. Commun..

[63] Wressnigg N., Hochreiter R., Zoihsl O., Fritzer A., Bezay N., Klingler A. (2020). Single-shot live-attenuated chikungunya vaccine in healthy adults: a phase 1, randomised controlled trial. Lancet Infect. Dis..

[64] Schneider M., Narciso-Abraham M., Hadl S., McMahon R., Toepfer S., Fuchs U. (2023). Safety and immunogenicity of a single-shot live-attenuated chikungunya vaccine: a double-blind, multicentre, randomised, placebo-controlled, phase 3 trial. Lancet.

[65] Simone B., Lienert F. (2025). Post-authorisation experience and reported adverse events following use of a virus-like particle chikungunya vaccine, United States and Germany, up to August 2025. Euro Surveill..

[66] Bennett S.R., McCarty J.M., Ramanathan R., Mendy J., Richardson J.S., Smith J. (2022). Safety and immunogenicity of PXVX0317, an aluminium hydroxide-adjuvanted chikungunya virus-like particle vaccine: a randomised, double-blind, parallel-group, phase 2 trial. Lancet Infect. Dis..

[67] Raju S., Adams L.J., Earnest J.T., Warfield K., Vang L., Crowe J.E. (2023). A chikungunya virus-like particle vaccine induces broadly neutralizing and protective antibodies against alphaviruses in humans. Sci. Transl. Med..

[68] Reisinger E.C., Tschismarov R., Beubler E., Wiedermann U., Firbas C., Loebermann M. (2019). Immunogenicity, safety, and tolerability of the measles-vectored chikungunya virus vaccine MV-CHIK: a double-blind, randomised, placebo-controlled and active-controlled phase 2 trial. Lancet.

[69] Ramsauer K., Schwameis M., Firbas C., Mullner M., Putnak R.J., Thomas S.J. (2015). Immunogenicity, safety, and tolerability of a recombinant measles-virus-based chikungunya vaccine: a randomised, double-blind, placebo-controlled, active-comparator, first-in-man trial. Lancet Infect. Dis..

[70] Maure C., Khazhidinov K., Kang H., Auzenbergs M., Moyersoen P., Abbas K. (2024). Chikungunya vaccine development, challenges, and pathway toward public health impact. Vaccine.

[71] Shaw C.A., August A., Bart S., Booth P.J., Knightly C., Brasel T. (2023). A phase 1, randomized, placebo-controlled, dose-ranging study to evaluate the safety and immunogenicity of an mRNA-based chikungunya virus vaccine in healthy adults. Vaccine.

[72] Skalinski L.M., Santos A.E.S., Paixao E., Itaparica M., Barreto F., da Conceicao Nascimento Costa M. (2023). Chikungunya seroprevalence in population-based studies: a systematic review and meta-analysis. Arch. Public Health.

[73] Bustos Carrillo F., Collado D., Sanchez N., Ojeda S., Lopez Mercado B., Burger-Calderon R. (2019). Epidemiological evidence for lineage-specific differences in the risk of inapparent chikungunya virus infection. J. Virol..

[74] Montenegro-Quinonez C.A., Louis V.R., Horstick O., Velayudhan R., Dambach P., Runge-Ranzinger S. (2023). Interventions against aedes/dengue at the household level: a systematic review and meta-analysis. EBioMedicine.

[75] Pinto S.B., Riback T.I.S., Sylvestre G., Costa G., Peixoto J., Dias F.B.S. (2021). Effectiveness of Wolbachia-infected mosquito deployments in reducing the incidence of dengue and other Aedes-borne diseases in Niteroi, Brazil: a quasi-experimental study. PLoS Negl. Trop. Dis..

[76] Ribeiro Dos Santos G., Durovni B., Saraceni V., Souza Riback T.I., Pinto S.B., Anders K.L. (2022). Estimating the effect of the wMel release programme on the incidence of dengue and chikungunya in Rio de Janeiro, Brazil: a spatiotemporal modelling study. Lancet Infect. Dis..

[77] Maciel-de-Freitas R., Sauer F.G., Kliemke K., Garcia G.A., Pavan M.G., David M.R. (2024). Wolbachia strains wMel and wAlbB differentially affect *Aedes aegypti* traits related to fecundity. Microbiol. Spectrum.

